# In-Line Diffuse Reflectance Spectroscopy Enables Rapid Monitoring of Full-Scale Anaerobic Co-Digestion

**DOI:** 10.1021/acs.energyfuels.5c04529

**Published:** 2025-11-20

**Authors:** Zoe A. M. Kramin, Maclaine K. Putney, Craig L. Just

**Affiliations:** † IIHR – Hydroscience and Engineering, University of Iowa, Iowa City, Iowa 52242, United States; ‡ Department of Civil & Environmental Engineering, University of Iowa, Iowa City, Iowa 52242, United States

## Abstract

In-line diffuse reflectance spectroscopy with partial least-squares regression was piloted at a full-scale municipal anaerobic codigestion facility to evaluate rapid monitoring of heterogeneous feedstocks and digestate. Models developed from 42 high-strength waste samples and 146 digestate samples yielded operationally useful predictions (root-mean-square error 10–27%) for total solids, volatile solids, fats, chemical oxygen demand, volatile acids, and alkalinity. Protein and carbohydrate predictions were less accurate due to limitations in reference data. Importantly, predicted volatile acid:alkalinity ratios provided rapid indicators of digester stability. Downsampling analysis demonstrated that effective models could be developed with fewer than 50 training samples for several parameters, highlighting opportunities to reduce analytical costs. Field deployment during periods of digester instability, including foaming and failure, further validated the robustness of diffuse reflectance spectroscopy models under dynamic operating conditions. These results establish diffuse reflectance spectroscopy as a potentially cost-effective tool for improving process stability, biogas yield, and decision-making at full-scale anaerobic codigestion facilities.

## Graphical abstract

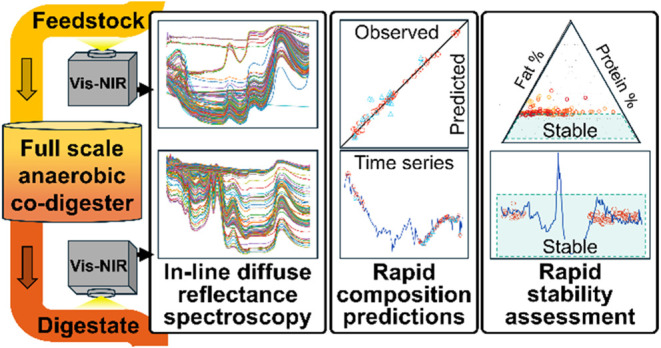

## Introduction

Anaerobic digestion (AD) is a biological process that breaks down organic materials to produce both biosolids suitable for agricultural use and bioenergy. Anaerobic digesters capture energy from organic feedstock degradation in the form of biogas that is rich in methane, the primary component of natural gas. Common feedstocks include municipal sludge and livestock manure, while energy crops and food waste can also be added through a process known as anaerobic codigestion (AcoD). Anaerobic digesters in the United States are underutilized as they can process 43 million tons of food waste annually, yet only 41% of this capacity was used in 2019.^1^ This excess capacity could be leveraged to process the 66 million tons of food waste generated annually in the U.S., diverting it from landfills where decomposing organic waste releases methane, a potent greenhouse gas that contributes significantly to global emissions.^1,2^ Incorporating more heterogeneous feedstocks, such as food waste, via AcoD should be encouraged to reduce landfill burden, lower greenhouse gas emissions, and enhance renewable energy production.

Maximizing biogas production from increasingly heterogeneous feedstocks while maintaining stable AcoD operations requires frequent monitoring of key physicochemical parameters to prevent process disruptions.^3^ Digester upsets are commonly triggered by organic overloading and resulting pH drops, as well as excess protein degradation, which can lead to biological inhibition due to ammonia accumulation.^3,4^ Organic loading rate (OLR) is determined by the volatile solids (VS) content of the feedstock in combination with its flow rate.^5,6^ VS loading is typically between 0.5 and 1.6 kg of VS per cubic meter per day (kg VS m^–3^ d^–1^) and 1.6 and 6.4 kg VS m^–3^ d^–1^ for standard rate and high rate AD, respectively.^7^ Beyond quantity, understanding the composition of organics is essential, as it significantly influences AcoD performance. Carbohydrate-rich feedstocks (e.g., vegetables) digest rapidly and with minimal inhibition, whereas fat-rich feedstocks (e.g., oils) degrade more slowly and may release inhibitory organic acids, and protein-heavy feedstocks (e.g., fish) can cause complete inhibition due to high levels of ammoniacal nitrogen production.^8^ The ratio of fats, proteins, and carbohydrates in feedstock has proven to be a useful indicator of digester stability, with protein content exerting the greatest influence.^9^ The AcoD process is generally stable when the protein content of incoming feedstock is between 5–25%.^9^ In addition to feedstock characteristics, digester health is strongly indicated by internal conditions, particularly volatile acid (VA) and alkalinity levels.^5^ The total VA to alkalinity ratio (VA:Alk) is a valuable monitoring tool in AD because it signals when acid levels exceed the system’s buffering capacity, with established thresholds defining safe operating zones at ratios of 0.1 or less.^5^ These physicochemical parameter limits can act as guides to inform digester dosing.

In more aggressive AcoD applications, the physicochemical conditions of both the feedstock and digester can shift more rapidly than traditional monitoring programs are designed to oversee. Conventional AD monitoring protocols typically only recommend one daily measurement of key parameters like total solids (TS), VS, VA, alkalinity, and temperature, with chemical oxygen demand (COD) often tested only weekly.^10^ This limited frequency is inadequate for highly variable AcoD systems. Although more frequent grab sampling could provide greater insight, it is both time-consuming and costly, leading most facilities to operate within conservative OLRs.^11^ Furthermore, the key microbial populations responsible for AD can respond to shifts in feedstock loading and composition within hours,^12,13^ emphasizing the need for faster monitoring approaches. Access to rapid, consistent monitoring of physicochemical parameters would empower operators to optimize performance and fully harness the biogas potential of AcoD systems.^11^

Diffuse reflectance spectroscopy (DRS), combined with machine learning, can deliver rapid physicochemical insights for feedstocks and digestate. DRS is an appealing tool because it minimizes the time, materials, and waste associated with traditional physicochemical laboratory analyses.^14^ DRS uses a light source and detector to interact with C–H, O–H, N–H, and S–H bonds, which are common in organic materials, producing diffusely backscattered light that enables wavelength-specific intensity detection.^15^ This makes DRS well-suited for heterogeneous, light-scattering organic samples and difficult applications such as in-line monitoring of nontransparent slurries.^15–17^ Machine learning methods can interpret diffuse reflectance spectra to predict the concentrations of constituents within these slurries. Partial least-squares regression (PLS) is the most commonly used predictive model for DRS.^18^ PLS projects latent variables in high-dimensional space to establish relationships between measured spectra and corresponding physicochemical properties.^18–21^ Modeling physicochemical concentrations in slurries using spectral data is challenging due to the strong influence of the water-to-dry matter ratio on absorption and reflection, but combining simple preprocessing methods like detrend and standard normal variate can reduce random and systematic variations by addressing additive and multiplicative water effects.^23^

The use of DRS for AD has been demonstrated at full- and lab-scales with satisfactory results, though rapid monitoring of incoming feedstocks and full-scale facilities is rare.^22^ The few studies that used DRS for rapid monitoring of parameters similar to those in our study were not applied to full-scale AcoD systems (Supporting Information (SI) Table S1).^23–30^ Accuracy will inherently be higher for models predicting parameters in highly controlled reactors, like at monodigestion facilities, because parameter concentrations will remain in a small range. Although dynamic operating conditions in full-scale AcoD systems may lower predictive accuracy, rapid models can remain operationally valuable despite high error (discrepancies up to 37%) by accurately predicting state of the AD process (e.g., stable or critical).^22,31^ Demonstrating the feasibility and utility of DRS under the dynamic conditions of full-scale AcoD operations is therefore critical to advancing its adoption as a practical tool for optimizing biogas production and process stability.

The objectives of this study were to conduct an in-line DRS pilot at a full-scale AcoD facility to inform operator decision-making by (1) generating rapid predictions of key physicochemical parameters in both feedstock and digestate; (2) applying these predictions to assess digester stability under dynamic operating conditions; and (3) determining the minimum reference data set size required to achieve acceptable prediction accuracy for each parameter.

## Materials and Methods

### Facility Information

This pilot-scale study was conducted at the Muscatine Water Resource Recovery Facility (WRRF) located in Muscatine, Iowa (population ∼24,000). The facility treats approximately 5.5 million gallons per day (gal d^–1^) and operates two mesophilic (35 C), floating-top, continuously stirred anaerobic digesters (∼485,000 gallons each) for the codigestion of primary sludge (PS), thickened waste activated sludge (TWAS), and hauled organics (SI Figure S1). Up to 20 tons per hour of hauled organics from food and beverage producers, residents, animal feed manufacturers, and grocery stores were depackaged at the Muscatine Organic Recycling Center using mechanical equipment (T42 Turbo Separator, Scott Equipment, Minnesota). The recovered organic slurry was transported by truck to the Muscatine WRRF and deposited into a continuously mixed tank (∼65,000 gallons), where it was combined with unpackaged organics from environmental waste management firms, food service establishments, and grease trap/interceptor cleaning services to form the high-strength waste (HSW) stream. During the study, the two digesters were daily fed approximately 7000 gallons of HSW, 25,000 gallons of PS, and 14,000 gallons of TWAS. The Muscatine WRRF annually produces approximately 4.5 million gallons of Class B biosolids, which are land applied to 550 acres of farmland in Muscatine County. All following mentions of digestate are referring to digestate from Digester 1.

### Grab-Sample Collection and Analysis

Grab samples of HSW and digestate were analyzed from December 20, 2023, to May 31, 2024, to train and test predictive models. HSW samples (*n* = 42, no data removed) were collected in 1-L bottles (Nalgene) pretreated with three milliliters of sulfuric acid. The HSW samples were analyzed for volatile acids (VA as acetic acid, in milligrams per liter (mg L^–1^)) and COD (mg L^–1^) using Hach TNT 872 and TNT 823 kits, respectively. Samples were diluted 1:100 and stirred before digestion in a heating unit (DRB200, Hach, Colorado) and spectrophotometric analysis (DR3900, Hach). TS (mg L^–1^, in triplicate) were determined using an infrared moisture and solids analyzer (Smart6, CEM, North Carolina) and VS (mg L^–1^, in triplicate) were determined by weight difference after combustion in a microwave muffle furnace (Phoenix Black, CEM) at 650 °C for 10 min. Fat content (Fat, as %TS, in triplicate) was determined using the Smart6 infrared moisture and solids analyzer. Crude protein content (Protein, as %TS) of HSW samples was determined via combustion (AOAC 990.03, Midwest Laboratories, Nebraska). Carbohydrate content (Carbs) was estimated by difference as carbohydrate (%) = 100% – Fat (%) – Protein (%), which is a commonly used yet limited empirical method for carbohydrate proximate analysis.^32^ This approach assumes that all measured solids are composed exclusively of protein, fat, and carbohydrate, and does not explicitly account for other solids such as ash, fiber, or plastic, leading to overestimations of carbohydrate content.^32,33^ Despite these limitations, this proxy estimates carbohydrate content without expensive carbohydrate fraction analysis and allows for consistent ternary analysis among HSW samples.

The data set describing digestate physicochemical parameters (*n* = 146, no data removed), additional HSW TS and VS values (*n* = 80, *n* = 79, respectively), and daily loading of HSW, TWAS, and PS, was provided by the Muscatine WRRF laboratory staff. Parameters analyzed by the Muscatine WRRF laboratory included TS (2540 B Standard Methods, dried at 103–105 °C),^34^ VS (2540 E Standard Methods, ignited at 500 °C),^34^ and an automatic titrator (HI 901, Hanna Instruments, Rhode Island) was used to determine VA and alkalinity via end point titration to pH 4.4 and pH 5.0, respectively. All measurements were reported as mg L^–1^. During the sampling period, the Muscatine WRRF laboratory was determining VA and alkalinity concentrations from both whole digestate samples (indicated by VA_whole_ and Alk_whole_) and from digestate supernatant (indicated by VA_supernatant_ and Alk_supernatant_) to compare titration results. Supernatant was collected from digestate after centrifuging samples for 5 min at 2000 rotations per minute. Some samples underwent a partial physicochemical analysis due to logistical and operational constraints; therefore, the sample number for model training and testing varied by target parameter.

### In-Line Diffuse Reflectance Spectroscopy

Mobile carts (Figure 1, SI Figure S2) containing a peristaltic pump (Peripro 10.7, Netzsch Pumps, Pennsylvania), a sensor mounting flange (X-Cell DN50, 2-in. diameter, Buchi Corporation, Delaware), and a spectrophotometer (NIR-Online X-Three, Buchi Corporation) were utilized for DRS of the HSW and digestate. These components were connected via PVC pipe (schedule 40), and the sensor flange was oriented vertically to ensure full-pipe measurements.

**Figure 1:**
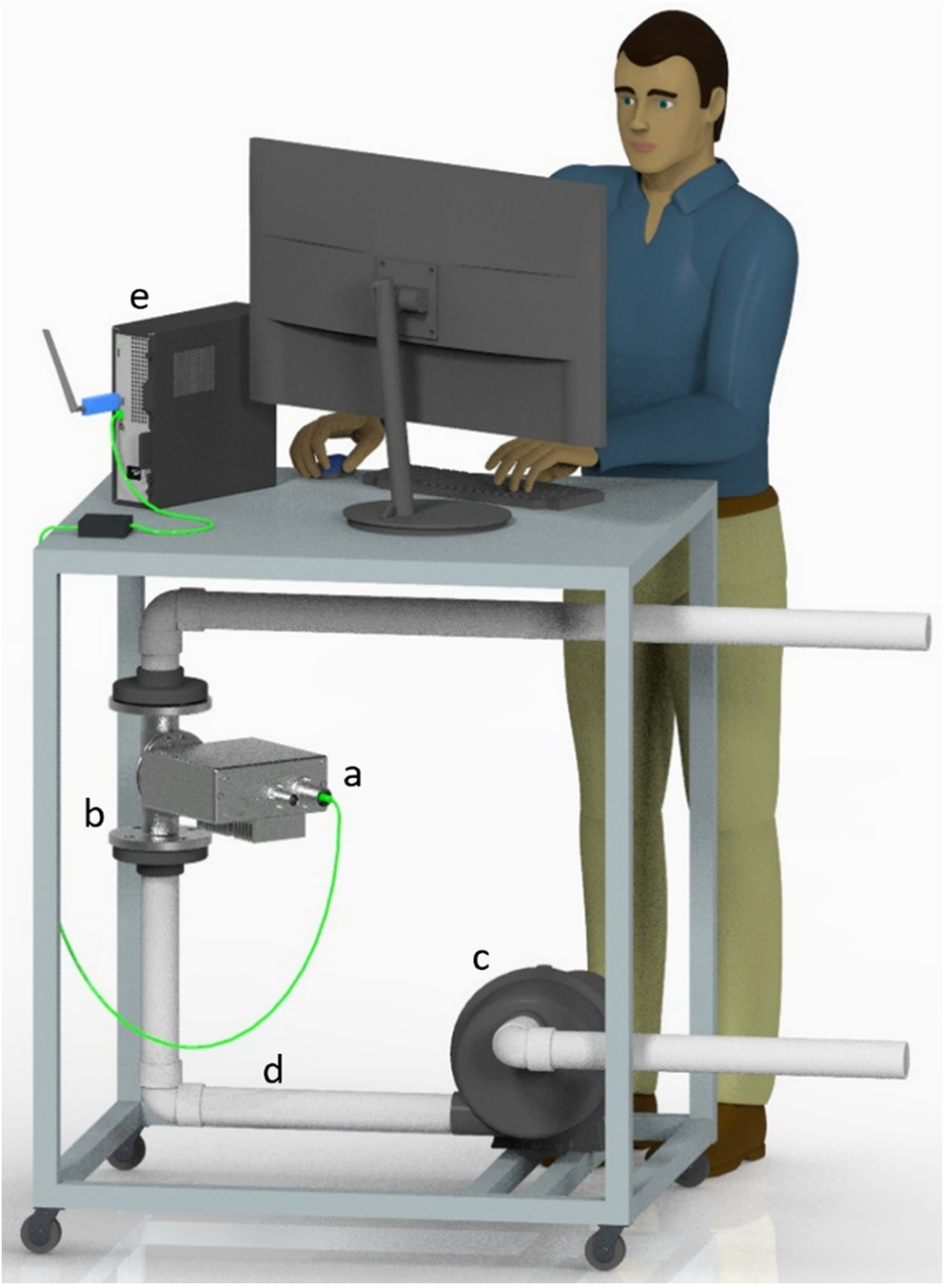
Illustrated representation of the mobile cart used for diffuse reflectance spectroscopy showing (a) spectrophotometer, (b) sensor mounting flange, (c) peristaltic pump, (d) PVC connection piping, and (e) personal computer.

The peristaltic pump maintained a stable flow (1.8 gallons per minute) of each waste stream. Digestate was routed to the mobile cart from the digestate recirculation pipe (6-in.) and HSW was routed from the HSW dosing pipe (4-in.). The HSW dosing and digestate recirculation pipes were each equipped with two nipples (1.5-in.) with ball valves that provided influent and effluent connection points for the mobile carts. The digestate cart was equipped with hoses (1.5-in. clear PVC suction hose, Stutsman Incorporated, Iowa) connected to the nipples via lockable fittings (1.5-in. stainless steel, Camlock Fittings, California). The HSW cart was identical to the digestate cart except that chemical resistant connection hoses (1.5-in. Prospector Pro Continental Hose, Stutsman Incorporated) were used. Additional nipples were used for grab-sample collection. The diffuse reflectance spectrophotometers were wired to a personal computer (Windows 10, Dell) running the control software (SX-Suite, Buchi Corporation). The spectrophotometers were configured for a wavelength range of 400 to 1700 nm with a 5 nm resolution (i.e., 261 spectral variables). Spectra were recorded each minute from December 20th, 2023, to May 31st, 2024, and the raw data were stored as JCAMP files.

### Model Training

A machine learning pipeline was developed in Python (v2024.14.1) to predict physicochemical concentrations from spectral data. Model development began with filtering training files for each waste stream to retain only data points with both grab-sample measurements and corresponding spectra. For each parameter, the script first checked whether at least ten samples were available for model training. If this criterion was met, the spectra were preprocessed using a Whittaker smoothing filter (derivative = 2, smoothing constraint order = 3) implemented using the *chemometrics* package, and then standardized using *StandardScaler* from *sklearn*.^35,36^

Concentration predictions were made using *PLSRegression* from *sklearn* with 5-fold cross-validation.^35^ The number of latent variables was varied from 1 to 10, and the optimal number was selected based on the lowest median cross-validation root-mean-square error (RMSE) across folds. Too many latent variables can lead to overfit models, but this is avoided by using the cross-validation RMSE values to determine the optimal number of latent variables.^37^ This final model was then used to compute performance metrics and to generate predictions every minute to provide near real-time insights.

### Model Testing

For the highly variable HSW, the exact time of each grab sample was recorded to select the corresponding spectrum with the closest timestamp. For the more stable digestate, a single spectrum was consistently extracted on sampling days to align with the standard grab-sample time of 8:30 AM. The Kennard-Stone algorithm was used to partition samples into training and testing sets based on the spatial distribution in feature space, ensuring a uniform and representative selection, which is an especially important consideration when working with limited data.^38^ A 70/30 train/test split was applied. Models were developed using the physicochemical and spectral data from the training set, then predicted concentrations based on spectral variables. The testing set was reserved exclusively for model evaluation and was not used during model training. Each model pipeline was evaluated using correlation coefficients (*R* ^2^, SI eq S1), percent root-mean-square error (RMSE%, SI eq S2), and residual prediction deviation (RPD, SI eq S3).

### Downsampling Analysis

Downsampling was conducted to assess model performance when trained on progressively smaller data sets. Each physicochemical parameter data set was progressively reduced based on the total number of observations, ultimately reaching a minimum subset of 20 data points. Larger data sets (i.e., TS and VS) were reduced by increments of ten observations, whereas smaller data sets were reduced by increments of five. To reduce variability introduced by random selection, each downsampling reduction scenario was repeated 20 times using random seeds from 0 to 19, and model performance metrics were averaged across repetitions. After each subset was generated, models were built and evaluated following the procedures described above, enabling direct comparison of performance across the full and reduced data sets.

## Results and Discussion

### HSW Modeling

Model evaluation criteria depend on the intended application. In general, models are considered statistically insufficient when *R* ^2^ < 0.6 and RPD < 1.4.^39,40^ However, models with a low *R* ^2^ and RPD can still be useful for practical applications if the prediction RMSE remains low.^39,41^ Therefore, reducing RMSE to an acceptable threshold is crucial for models being applied to full-scale AcoD, and should be prioritized over *R* ^2^ and RPD. While models with RMSEs below 50% may still reflect overall data patterns,^42,43^ predictive accuracy is generally considered inadequate when RMSE exceeds 30%.^44^ RMSEs below 30 and 15% are typically regarded as acceptable and strong, respectively.^43–45^ Accordingly, models resulting in RMSEs below 30% were deemed satisfactory for operational use in this study.

In particular, the VS model for HSW was operationally valuable as it performed under the RMSE% threshold. Given its common use as an operational parameter for AD, the HSW OLR (kg VS m^–3^ d^–1^) was modeled using predicted VS. Predictions were visualized with a time series and one-to-one plot, and evaluated using RMSE%, *R* ^2^, and RPD, which demonstrates that the model could have identified periods of OLR exceedances in near real-time, on a minute-by-minute basis (Figure 2a). The total OLR, derived from the combined HSW, PS, and TWAS streams (Figure 2b), frequently exceeded the standard OLR range of 0.5–1.6 kg VS m^–3^ d^–1^;^7,46^ however, these exceedances were predictable based on the DRS of the HSW (Figure 2c), particularly during periods when the observed OLRs for PS and TWAS showed little variation (Figure 2d). These results correspond with operator observations that the Muscatine WRRF was processing highly variable HSW, presenting a potential risk of digester failure due to OLR exceedances and/or rapid fluctuations.

**Figure 2:**
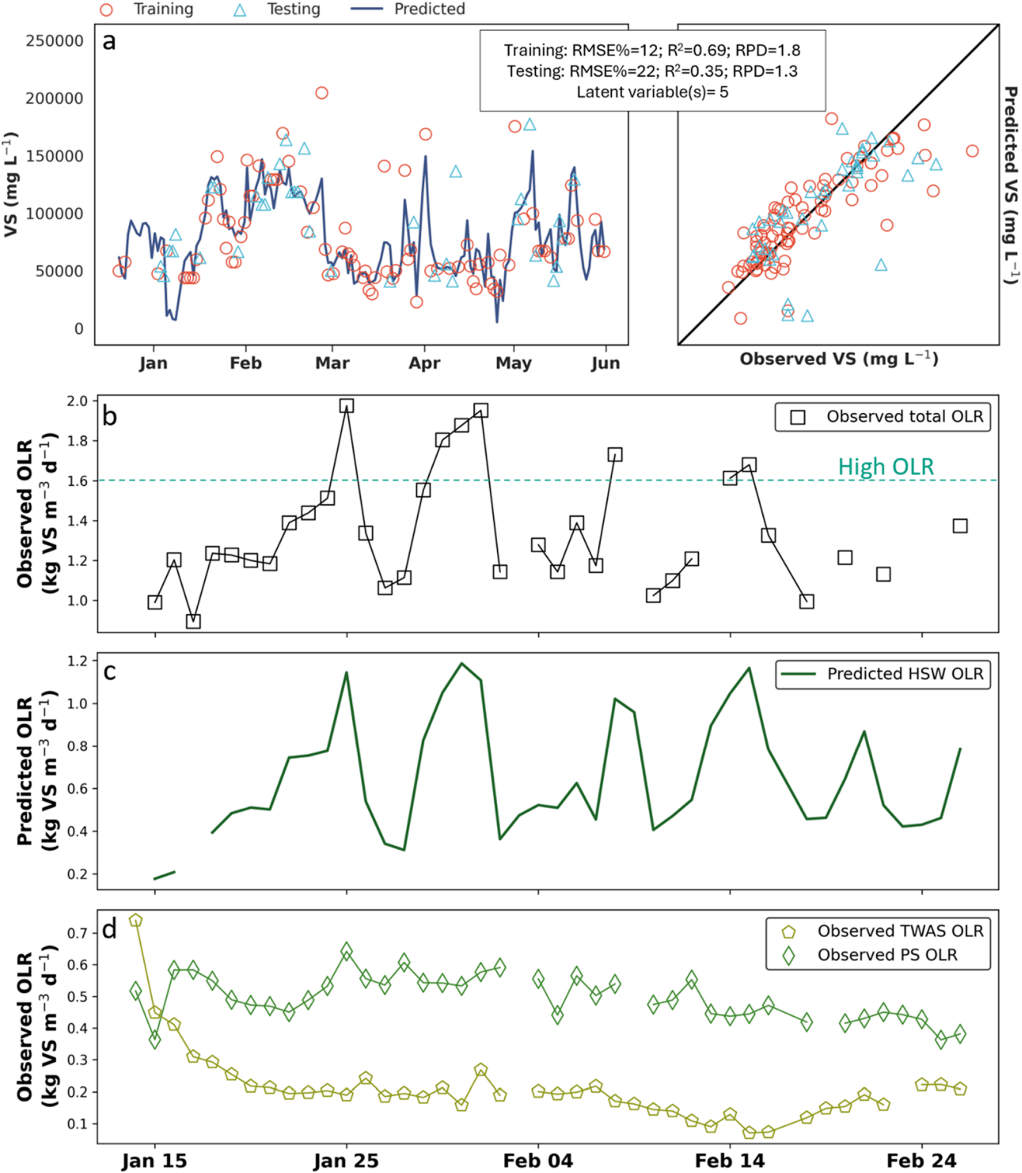
Predictions of VS in HSW applied to OLR. (a) Results of training and testing the model predicting VS in HSW over the sampling period (December 20, 2023, to May 31, 2024) and comparison of observations to predictions. The bisector line represents a *R* ^2^ of 1. (b) Observed total OLR from January 12 to February 28, calculated using observed VS concentration and flow rate values from HSW, PS, and TWAS. Dashed horizontal line represents high OLR defined as 1.6 kg VS m^–3^ d^–1^ or greater. (c) Predicted HSW OLR from January 12 to February 28, calculated using observed HSW flow rates and predicted HSW VS concentrations. (d) Observed PS and TWAS OLR from January 12 to February 28, calculated using observed concentration and flow rate values from PS, and TWAS. HSW, high-strength waste; mg L^–1^, milligrams per liter; OLR, organic loading rate; PS, primary sludge; TWAS, thickened waste activated sludge; VS, volatile solids; kg VS m^–3^ d^–1^, kilograms of VS per cubic meter per day; RMSE%, percent root-mean-square error; *R* ^2^, correlation coefficient; RPD, residual prediction deviation.

We also modeled fat, protein, and carbohydrate content in HSW, with particular emphasis on protein due to its potential to contribute to ammonia toxicity during AD. Like the results for VS, the DRS model for fat performed well enough to offer operational value (Figure 3a); however, the carbohydrate and protein models were not reliable. Protein predictions (Figure 3b) were skewed toward the observed mean of training values. This pattern is indicative of an oversimplified model that defaults to the baseline mean, potentially due to limitations in the chosen chemical analysis method. Although the carbohydrate model (Figure 3c) performed similarly to the fat model, it required more latent variables than protein and fat models, suggesting potential overfitting. Because carbohydrate content was calculated by difference, its predictive performance depended on the accuracy of fat and protein measurements, with the latter likely being compromised by methodological limitations.

**Figure 3:**
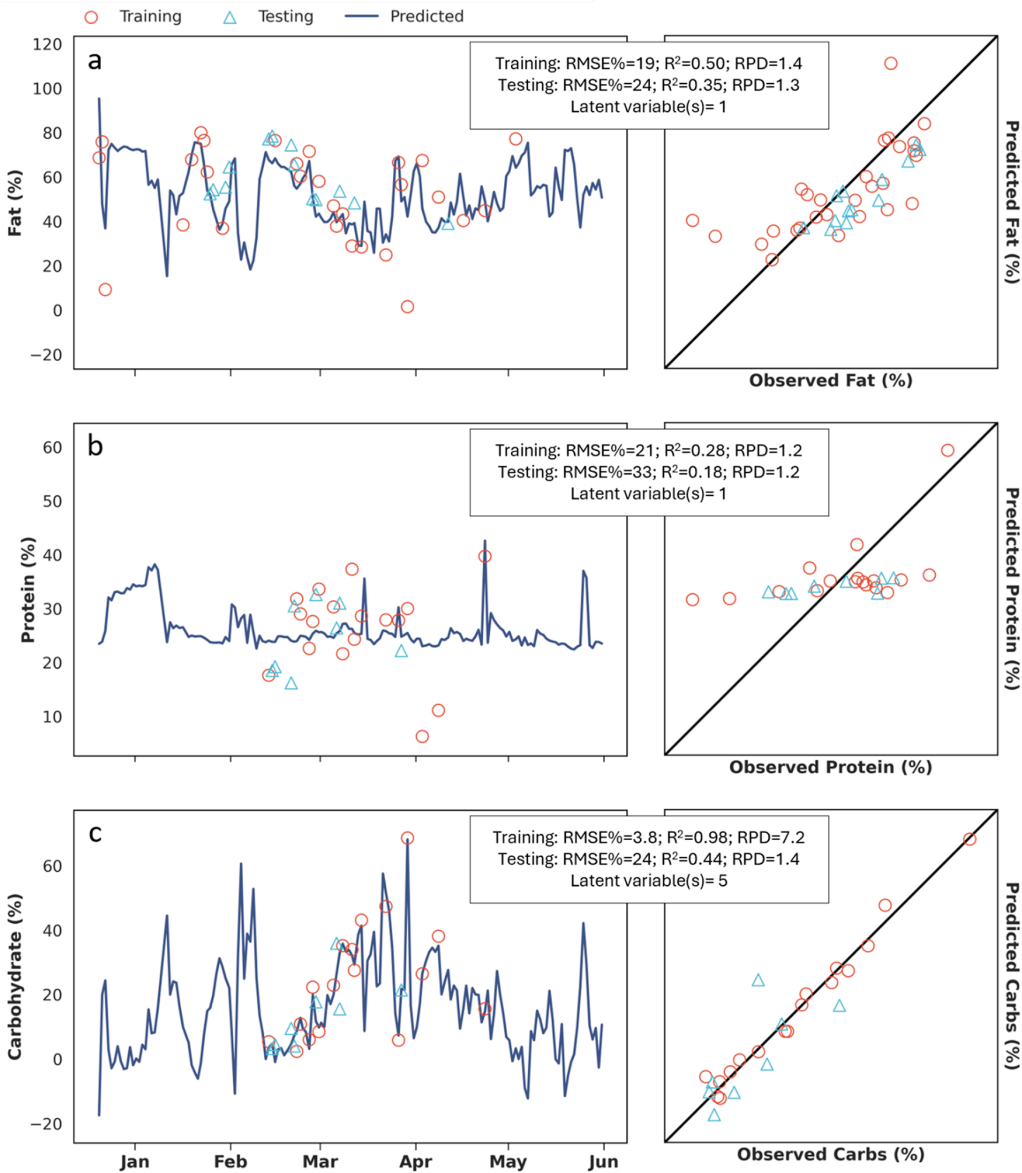
Predictions compared to observations of fat, protein, and carbohydrates in HSW. (a) Results of training and testing the model predicting fat in HSW over the sampling period (December 20, 2023, to May 31, 2024) and comparison of observations to predictions. (b) Results of training and testing the model predicting protein in HSW over the sampling and comparison of observations to predictions. (c) Results of training and testing the model predicting carbohydrates in HSW over the sampling and comparison of observations to predictions. The bisector line (a–c) represents a *R* ^2^ of 1. HSW, high-strength waste; Carbs, carbohydrates; RMSE%, percent root-mean-square error; *R* ^2^, correlation coefficient; RPD, residual prediction deviation.

Standard approaches for estimating crude protein, such as combustion (e.g., AOAC 990.03), apply a universal nitrogen conversion factor of 6.25, based on the assumption that proteins contain 16% nitrogen. While commonly used, this assumption is inaccurate and can introduce substantial errors.^47^ In reality, nitrogen content and conversion factors vary depending on the protein source (e.g., 4.94 for fish, 5.94 for milk).^48^ This variability poses a challenge when modeling heterogeneous waste streams, as the relative proportions of different protein sources fluctuate over time. For homogeneous feedstocks (e.g., maize silage), using fixed protein content factors can still yield reasonably accurate models, although with systematic errors, because the biochemical composition remains consistent. However, applying fixed protein content factors to diverse heterogeneous feedstocks (e.g., HSW) introduces random errors into the reference data set. Models trained on reference data sets with random errors have less predictive power. Therefore, we recommend using higher-precision methods, such as direct amino acid analysis, to establish a more accurate reference data set for modeling protein and carbohydrates in heterogeneous waste streams. A study monitoring incoming maize silage also used rapid predictive modeling to estimate fat and protein content.^26^ Although the models outperformed ours, the use of homogeneous feedstock, larger training data sets, and narrower parameter ranges potentially contributed in boosting model performance (SI Tables S1 and S2). This highlights the potential of DRS to model protein content, despite our study’s limitations related to heterogeneous feedstocks and a small training data set.

Mindful of the potential for ammonia toxicity from protein overloading, and despite current limitations in protein and carbohydrate modeling, this study aimed to demonstrate the operational value of near real-time predictions of fat, protein, and carbohydrate content. Grab-sample results for fat, protein, and carbohydrates in the HSW showed that the Muscatine WRRF frequently loaded protein levels exceeding 25% (Figure 4a). Protein levels outside the 5–25% range have been linked to process instability, as protein is the primary nitrogen source in the digester and plays a critical role in determining the carbon-to-nitrogen ratio.^9^ Our models accurately predicted fat percentages across a wide range, but protein predictions were skewed toward the observed mean of training values (26.3%), clustering near the upper limit of the stable range (Figure 4b). Despite the challenges of building reference data sets, the highly variable HSW loading and composition enabled successful modeling of complex parameters such as VS and fat, as well as TS, COD, and VA (SI Figure S4), offering valuable insights for full-scale facility operators.

**Figure 4:**
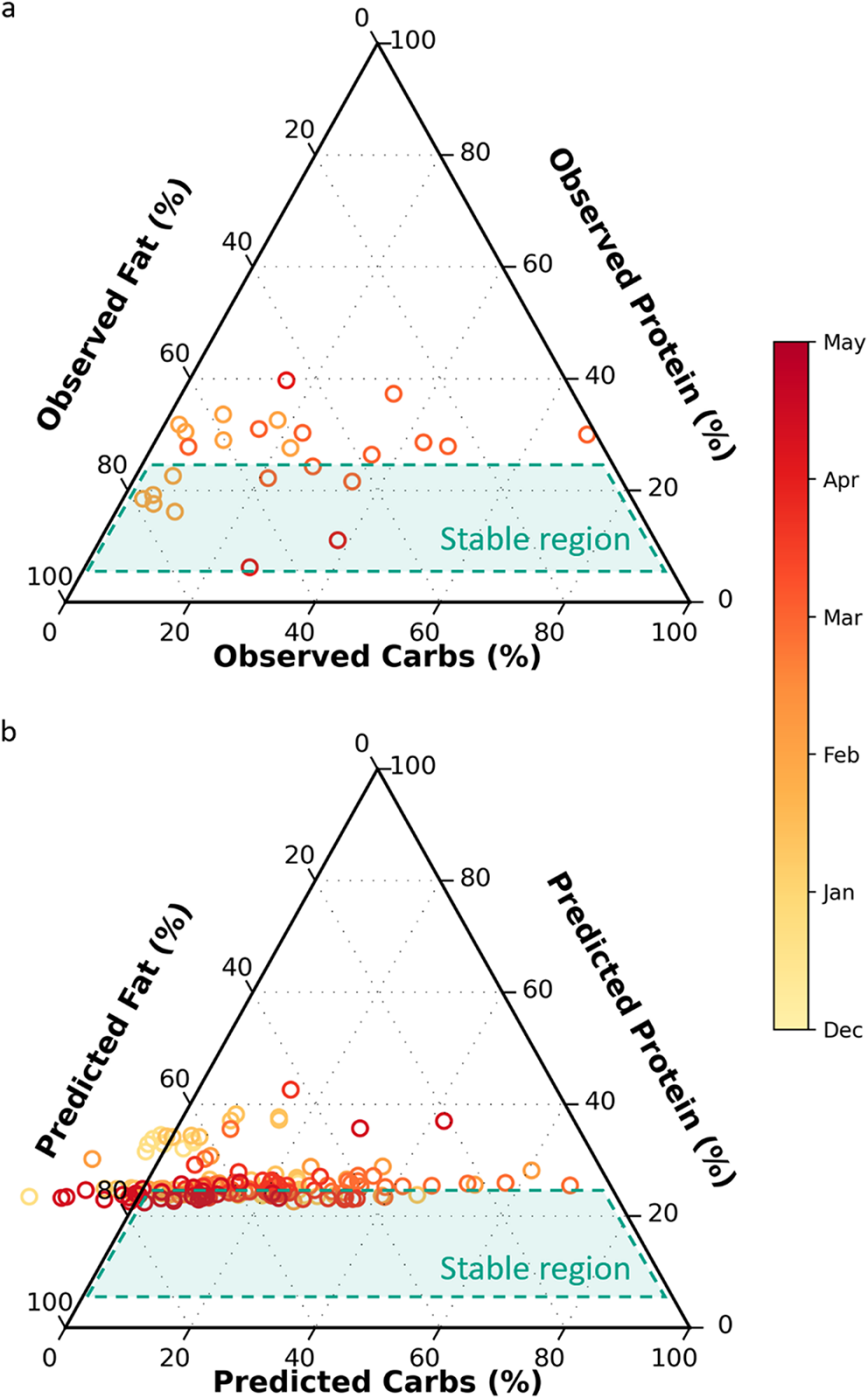
Ternary diagrams of fat, protein, carbohydrate ratio for observed (a) and predicted (b) values over the sampling period (December 20, 2023, to May 31, 2024). Dashed region represents stable digestion described by Cook et al. based on feedstock protein content (5–25%).^9^ Carbs, carbohydrates.

### Digestate Modeling

In monitoring digestate within the AD reactor, VA and alkalinity were frequently measured from either the supernatant or whole samples. For supernatant samples, our DRS models performed well enough to be highly useful for operators, as demonstrated by the time series and one-to-one plots, as well as good performance metrics, RMSE% ranging from 1.5 to 15%, *R* ^2^ values from 0.59 to 1, and RPD values between 1.6 and 19 (Figure 5, panels a and b). In contrast, the DRS model for VA_whole_ performed poorly (Figure 5c), whereas the Alk_whole_ model showed acceptable performance (Figure 5d). Suspended solids, oily residues, and other particulate matter present in whole samples can interfere with titration by coating electrodes or impeding chemical reactions,^34^ potentially reducing measurement accuracy. This may help to explain why the VA_supernatant_ reference data set produced more accurate predictions than the VA_whole_ model. Similarly, suspended solids and particulate matter interfere with VA photometric evaluation (e.g., Hach TNT 872) by scattering light. This interference likely contributed to the performance of the HSW VA model, which yielded a *R* ^2^ of 0, as supernatant was not collected from HSW samples for the reference data set. Among the models developed, the Alk_supernatant_ model exhibited the highest performance, possibly due to the increased complexity resulting from a high number of selected latent variables.^37^ The optimal number of latent variables selected for the alkalinity model was 10, the maximum permitted in our study, which aligns with findings from another DRS study on digester alkalinity, where 10 latent variables were also optimal despite an allowance of 15.^49^ We limited our models to 10 latent variables due to the small size of some data sets, as the number of latent variables must not exceed the number of training data points.^50^

**Figure 5:**
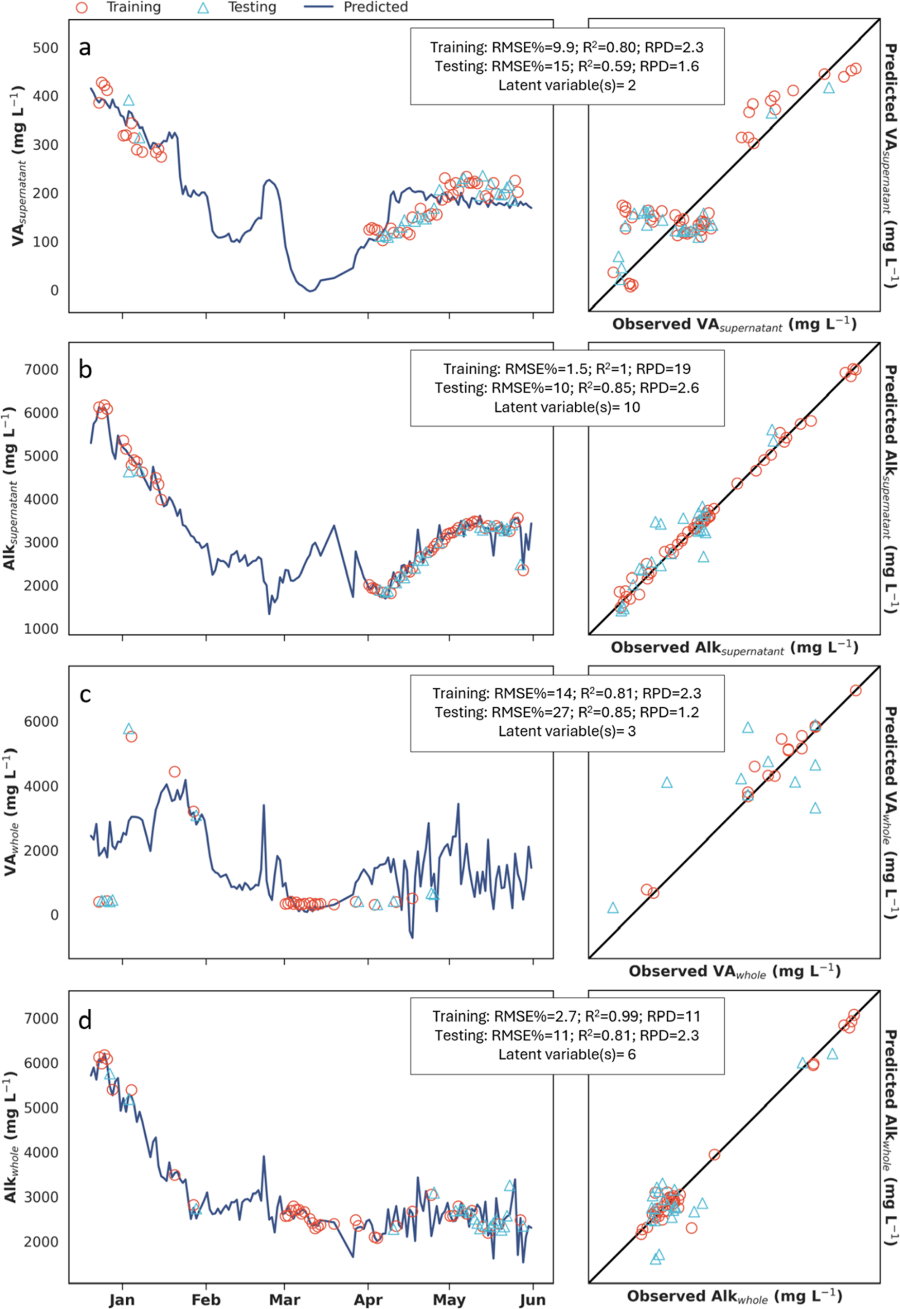
Predictions compared to observations of volatile acids and alkalinity in digestate over the sampling period (December 20, 2023, to May 31, 2024). (a) Results of training and testing the model predicting VA_supernatant_ over time and comparison of observations to predictions. (b) Results of training and testing the model predicting Alk_supernatant_ over time and comparison of observations to predictions. (c) Results of training and testing the model predicting VA_whole_ over time and comparison of observations to predictions. (d) Results of training and testing the model predicting Alk_whole_ over time and comparison of observations to predictions. The bisector line (a–d) represents a *R* ^2^ of 1. VA_supernatant_, volatile acids from digestate supernatant; Alk_supernatant_, alkalinity from digestate supernatant; VA_whole_, volatile acids from whole digestate sample; Alk_whole_, alkalinity from whole digestate sample; mg L^–1^, milligrams per liter; RMSE%, percent root-mean-square error; *R* ^2^, correlation coefficient; RPD, residual prediction deviation.

The balance between acidity and buffering capacity in a digester is a useful indicator because excess volatile acid production can deplete alkalinity, lowering pH and harming pH-sensitive methanogens. Operators often assess this balance using recommended ratios, with safe operation generally assumed to be below ratios of 0.1 and 0.4 for VA:Alk from supernatant and whole samples, respectively.^5,51^ Predicted VA and alkalinity concentrations can be used to calculate this ratio in near real-time, enabling comparison to target ranges for digester stability (Figure 6). This capability enables operators to intervene before instability escalates, changing digester management from reactive to predictive.

**Figure 6:**
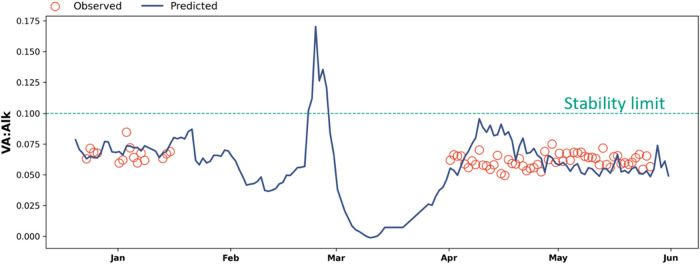
Predictions compared to observations of the volatile acid to alkalinity ratio in digestate supernatant (VA:Alk) over the sampling period (December 20, 2023, to May 31, 2024). Dashed horizontal line indicates an upper stability limit of 0.1.

### Downsampling

Typical model standards recommend 60–120 training samples or at least six times the number of latent variables.^52,53^ Given the time, equipment, and cost required to build reference data sets, full-scale operations benefit from minimizing grab samples so we evaluated whether sample sizes could be reduced through downsampling analysis. HSW data sets were reduced to 20–120 total data points, depending on the number of available observations for each parameter. After downsampling, HSW parameters, including TS, VS, COD, VA, and Fat, were predicted with satisfactory RMSE% values. Median RMSEs were 27% (TS, 49 samples), 24% (VS, 42 samples), 29% (COD, 24 samples), 30% (VA, 17 samples), and 27% (Fat, 14 samples) (Figure 7).

**Figure 7:**
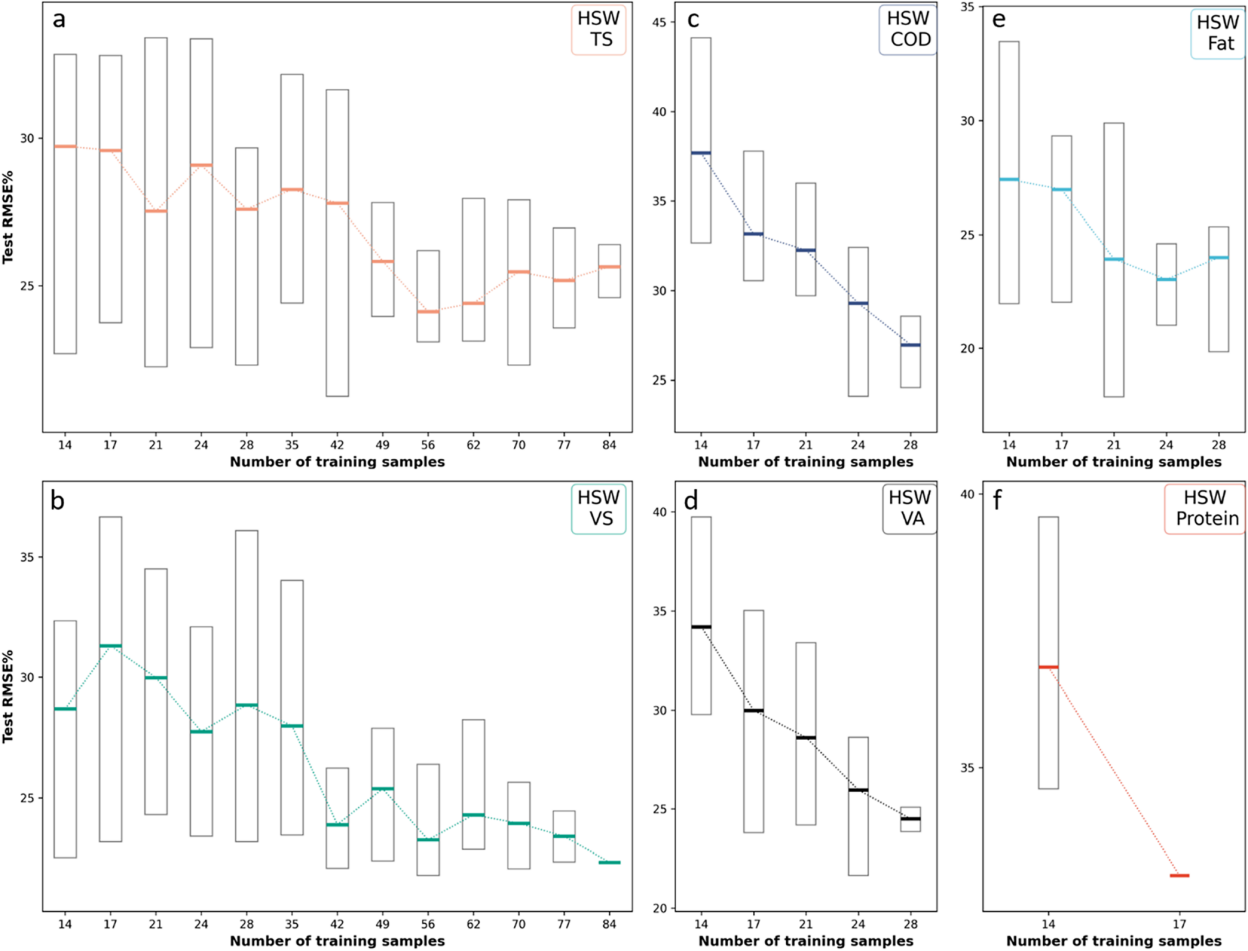
RMSE% summary of predicted parameters in HSW after downsampling. Each boxplot represents the distribution of RMSE% after 20 repetitions using the indicated number of training samples in each model. Horizontal line indicates the median while the edges of the boxes are the first and third quartiles. RMSE% summary of: (a) TS, (b) VS, (c) COD, (d) VA, (e) fat content, and (f) protein content. COD, chemical oxygen demand; HSW, high-strength waste; TS, total solids; VA, volatile acids; VS, volatile solids; RMSE%, percent root-mean-square error.

Digestate data sets were reduced to 20–140 data points, depending on the number of available observations for each parameter. Digestate parameters, including Alk_supernatant_, VA_supernatant_, and Alk_whole_, were also predicted with satisfactory RMSE% values after downsampling (Figure 8). Median RMSEs were 29% for Alk_supernatant_ (14 samples), 21% for VA_supernatant_ (21 samples), and 29% for Alk_whole_ (21 samples). In contrast, more variable parameters like TS and VS required larger data sets (training *n* = 98) to achieve RMSEs below 30%. These findings can help guide future sampling efforts at other facilities, reducing startup costs and accelerating deployment.

**Figure 8:**
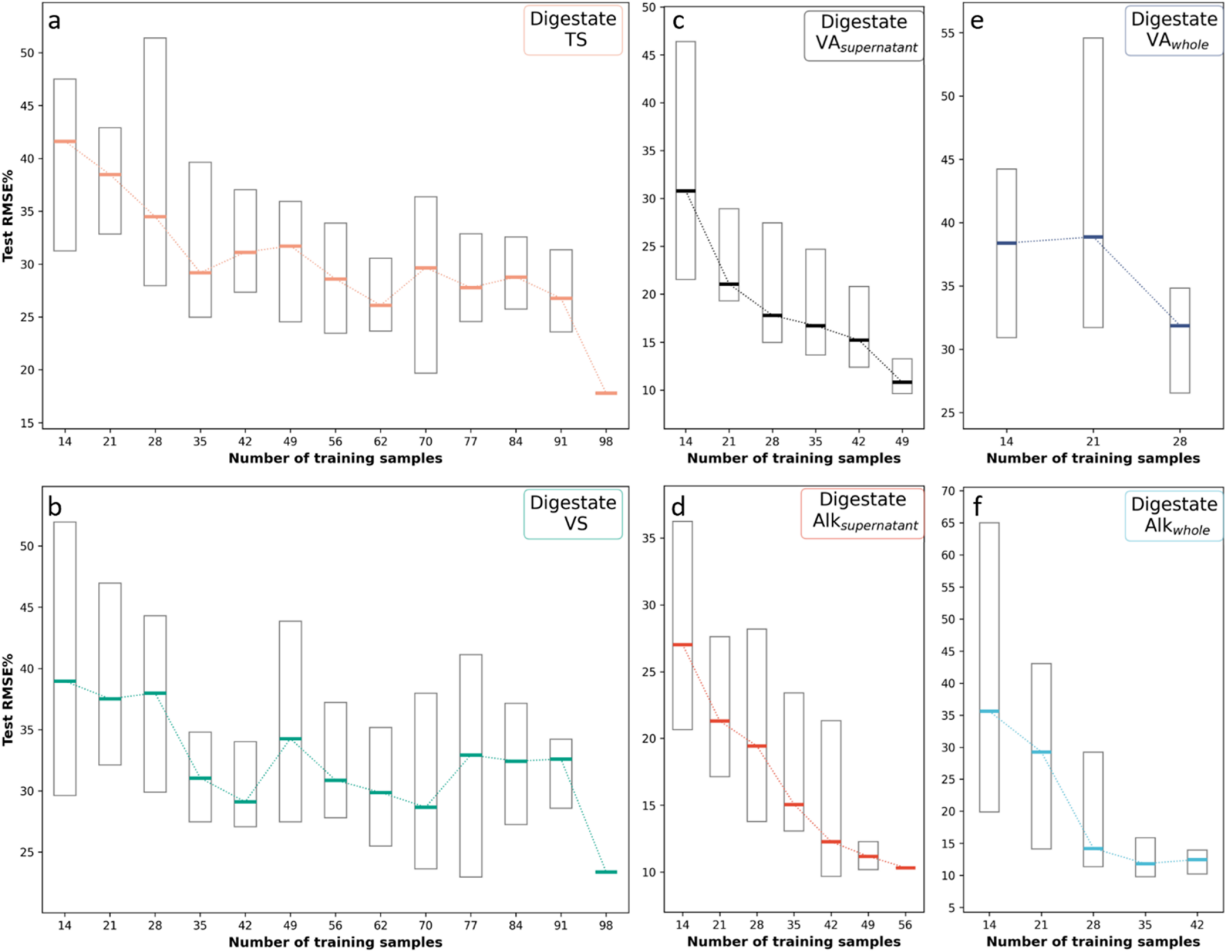
RMSE% summary of predicted parameters in digestate after downsampling. Each boxplot represents the distribution of RMSE% after 20 repetitions using the indicated number of training samples in each model. Horizontal line indicates the median while the edges of the boxes are the first and third quartiles. RMSE% summary of: (a) TS, (b) VS, (c) VA_supernatant_, (d) Alk_supernatant_, (e) VA_whole_, (f) Alk_whole_. TS, total solids; VS, volatile solids; Alk_supernatant_, alkalinity from digestate supernatant; VA_whole_, volatile acids from whole digestate sample; Alk_whole_, alkalinity from whole digestate sample; VA_supernatant_, volatile acids from digestate supernatant; RMSE%, percent root-mean-square error.

### Balancing Current Limitations with Financial Potential of DRS

Our field-deployed DRS models were trained on limited grab samples (*n* = 17–98) from a highly variable HSW stream during a period of instability at the Muscatine WRRF, which included operational disruptions such as a failure event in January, foaming episodes in February, and overloading during March. These operational extremes created a unique data set that enhances model robustness and offers rare insight into failure scenarios. Model performance may improve further by expanding training data, optimizing preprocessing, including wavelength selection, or incorporating operational variables (e.g., temperature, flow).

Despite slow adoption of DRS for anaerobic digestion due to concerns about equipment costs ($20k–100k) and model accuracy,^22^ the economic case is compelling. Biogas revenue is conservatively estimated at $0.05 per gallon of waste (eq 1), based on fixed assumptions for energy yield, electricity production, utility payment rates, and waste density.^54,55^ Under these conservative assumptions, a 20-day failure in 50,000 gal d^–1^ digesters could result in approximately $50,000 in lost biogas revenue. While the conservative estimate assumes utilities purchase electricity at approximately $0.03 per kilowatt-hour, WRRFs that generate electricity on-site could offset costs as high as $0.14 per kilowatt-hour, based on 2018 utility rates,^54^ suggesting a higher value for the cost benefit of biogas recovery. Additional income from feedstock tipping fees ($0.13–$0.26 per gallon of waste)^54,55^ further enhances the financial return of intensifying AcoD with high volumes of energy-dense feedstocks. Thus, the potential economic benefits of rapid DRS monitoring can outweigh its capital and operating costs.

1 $$\frac{3.5\;MMBtu}{ton\;of\;waste}*\frac{127\;kWh}{MMBtu}*\frac{\$0.03}{kWh}*\frac{ton}{2000\;lbs}*\frac{1500\;lbs}{{yd}^{3}}*\frac{yd^{3}}{201.974\;gallon}=\frac{\$0.05}{gallon\;of\;waste}$$

Equation 1. The potential biogas recovery cost benefit per gallon of waste. The fixed estimates of energy yield (MMBtu per ton), electricity production (kWh per MMBtu), and payment from utility (dollar per kWh) are from the Great Plains Institute (2018).^54^ The fixed estimate for the density of food waste (lbs per yd^3^) is from the EPA (2010).^55^ MMBtu, million British thermal units; kWh; kilowatt-hour; lbs, pounds; yd^3^, cubic yard.

## Conclusions

This study demonstrates that DRS, when paired with PLS, can provide operationally valuable predictions of both HSW and digestate characteristics in full-scale AcoD systems. Despite challenges related to sample heterogeneity, data set size, and modeling limitations, especially for protein, DRS models successfully predicted key parameters including TS, VS, COD, fat, alkalinity, and VA. Notably, downsampling analysis revealed that effective modeling can still be achieved with relatively few training samples for certain parameters, offering a cost-effective path forward for utilities considering DRS implementation. Furthermore, the ability to calculate near real-time VA:Alk ratios from model predictions supports more responsive and stable digester operation. The field deployment of DRS under conditions that included digester failure and recovery highlights the robustness and promise of this approach. When combined with potential revenue gains from biogas production and tipping fees, the economic case for DRS adoption is compelling, even in the face of capital and implementation costs. Continued refinement of spectral models and expansion of training data sets will further enhance predictive accuracy and operational value. Yet, the current models demonstrate strong predictive capability under full-scale AcoD conditions and represent a significant step forward in operational DRS monitoring, offering pathways to greater digester stability, higher biogas yields, and improved treatment performance.

## Supplementary Material



## Data Availability

The data and code underlying this study are openly available as “Dataset and Code for Rapid In-line Monitoring of Full-Scale Anaerobic Co-Digestion Using Diffuse Reflectance Spectroscopy and Machine Learning” in an open-source repository at DOI:10.25820/data.008042.

## Notes

Additional information not cited in the main text is provided in the SI; Figure S3 presents additional predictions of VS in HSW applied to OLR, while Figure S5 compares observed and predicted TS in digestate; Figure S6 shows predictions versus observations of the VA:Alk in whole digestate samples, and Figure S7 provides a summary of correlation coefficients for HSW parameters after downsampling; Table S2 summarizes the data used to train and test the partial least-squares regression model following the Kennard-Stone partition (PDF)
