# Supplementary material for: In-Line Diffuse Reflectance Spectroscopy Enables Rapid Monitoring of Full-Scale Anaerobic Co-Digestion
Source: Energy Fuels. 2025 Nov 20;39(48):22756–67. doi: 10.1021/acs.energyfuels.5c04529 (PMC12683639; doi:10.1021/acs.energyfuels.5c04529)
Supplement: Supplementary file 1 [file ef5c04529_si_001.pdf]

SUPPORTING INFORMATION

In-line Diffuse Reflectance Spectroscopy Enables Rapid Monitoring of Full-Scale Anaerobic Co-Digestion

*Zoe A.M. Kramin,‡ Maclaine K. Putney,‡ and Craig L. Just‡**

‡IIHR – Hydroscience and Engineering, University of Iowa, Iowa City, IA, USA, Department of Civil & Environmental Engineering, University of Iowa, Iowa City, IA, USA

*Email: craig-just@uiowa.edu

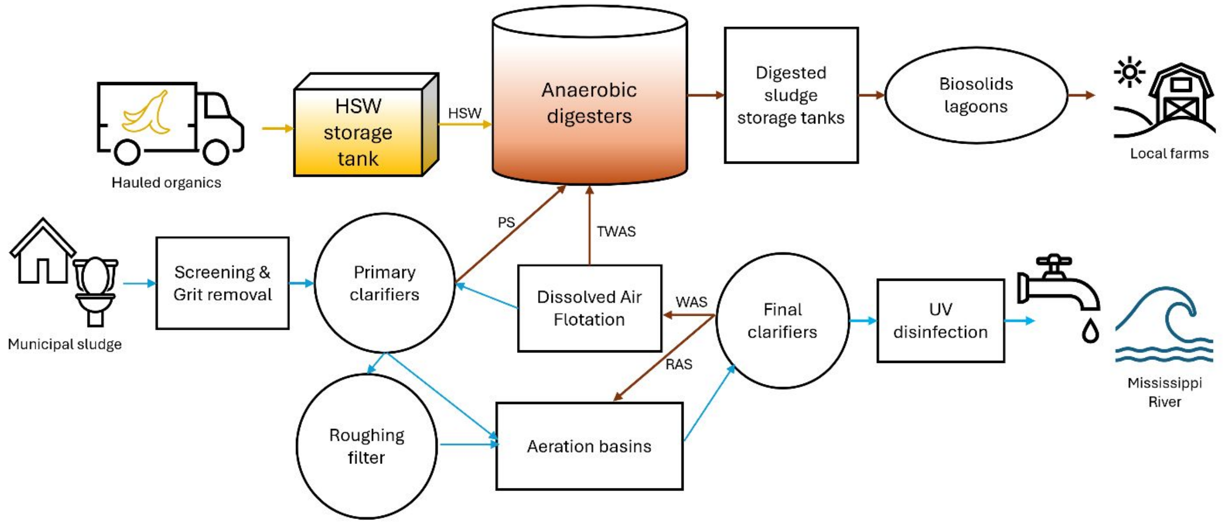

Figure S1. Process diagram of the Muscatine Water Resource Recovery Facility. HSW, high-strength waste; PS, primary solids; TWAS, thickened waste activated sludge; WAS, waste activated sludge; RAS, return activated sludge. Blue arrows indicate movement of liquid fraction, brown arrows indicate movement of solid fraction, yellow arrows indicate movement of HSW.

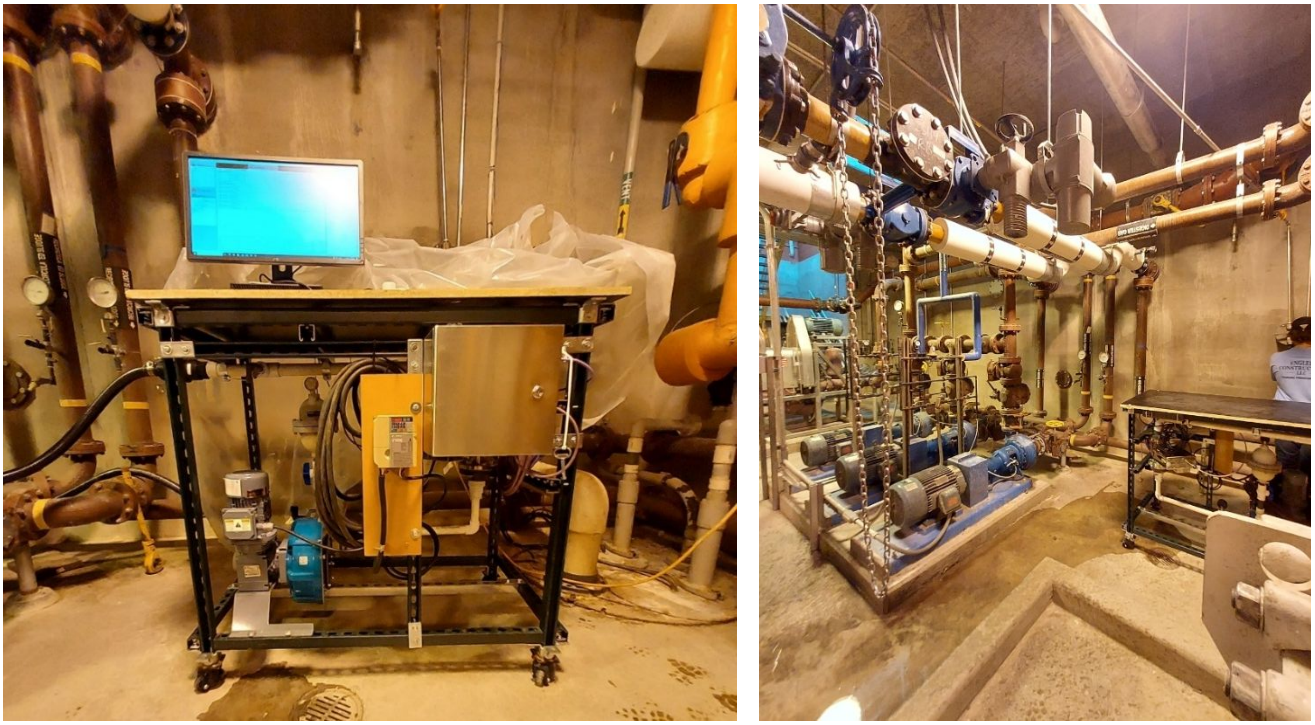

Figure S2. One mobile diffuse reflectance spectroscopy cart was deployed on the digestate stream at the Muscatine WRRF. A second cart was used for the high-strength waste stream nearby but is not shown here.

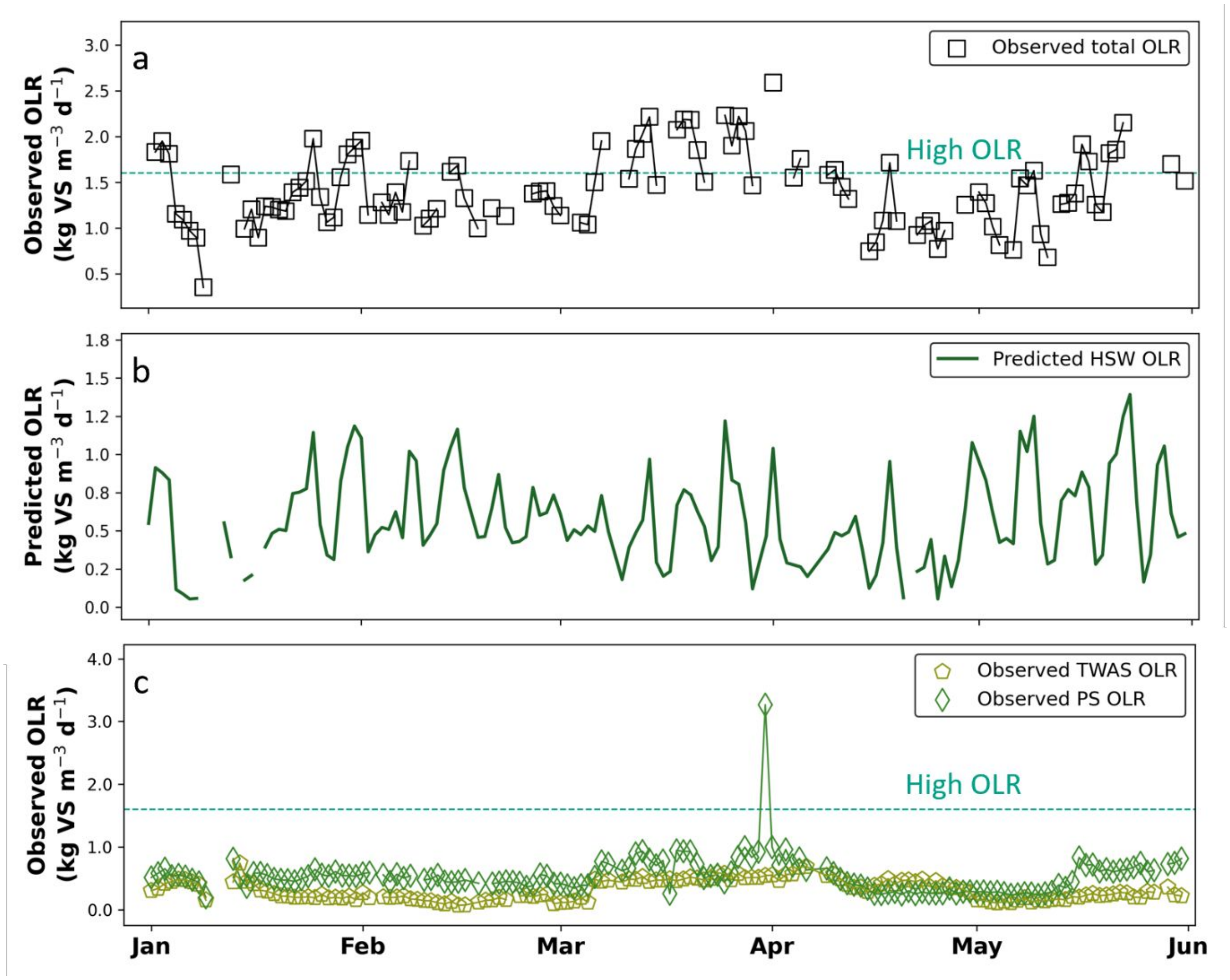

Figure S3. Predictions of VS in HSW applied to OLR. (a) Observed total OLR over the sampling period (December 20, 2023, to May 31, 2024), calculated using observed VS concentration and flow rate values from HSW, PS, and TWAS. Dashed horizontal line represents high OLR defined as 1.6 kg VS m^{-3} day^{-1} or greater. (b) Predicted HSW OLR over the sampling period, calculated using observed HSW flow rates and predicted HSW VS concentrations. (c) Observed PS and TWAS OLR over the sampling period, calculated using observed concentration and flow rate values from PS, and TWAS. HSW, high-strength waste; OLR, organic loading rate; PS, primary sludge; TWAS, thickened waste activated sludge; VS, volatile solids; kg VS m^{-3} day^{-1}, kilograms of VS per cubic meter per day.

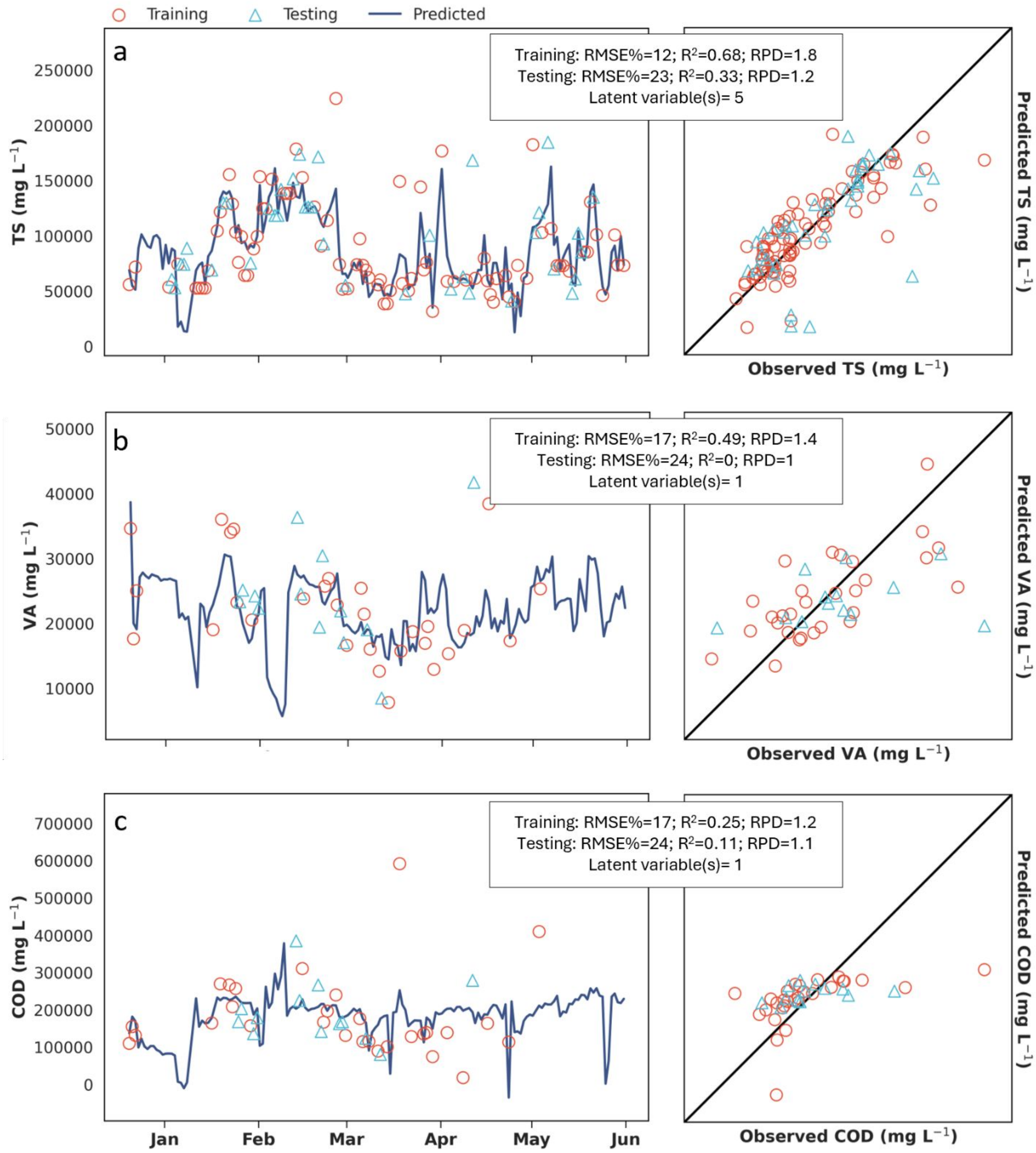

Figure S4. Predictions compared to observations of TS, VA, and COD in HSW. (a) Results of training and testing the model predicting TS in HSW over the sampling period (December 20, 2023, to May 31, 2024) and comparison of observations to predictions. (b) Results of training and testing the model predicting VA in HSW over the sampling period and comparison of observations to predictions. (c) Results of training and testing the model predicting COD in HSW over the sampling period and comparison of observations to predictions. The bisector line (a–c) represents a R^2 of 1. COD, chemical oxygen demand; HSW, high-strength waste; TS, total solids; VA, volatile acids; mg L^{-1}, milligrams per liter; RMSE%, percent root mean square error; R^2, correlation coefficient; RPD, residual prediction deviation.

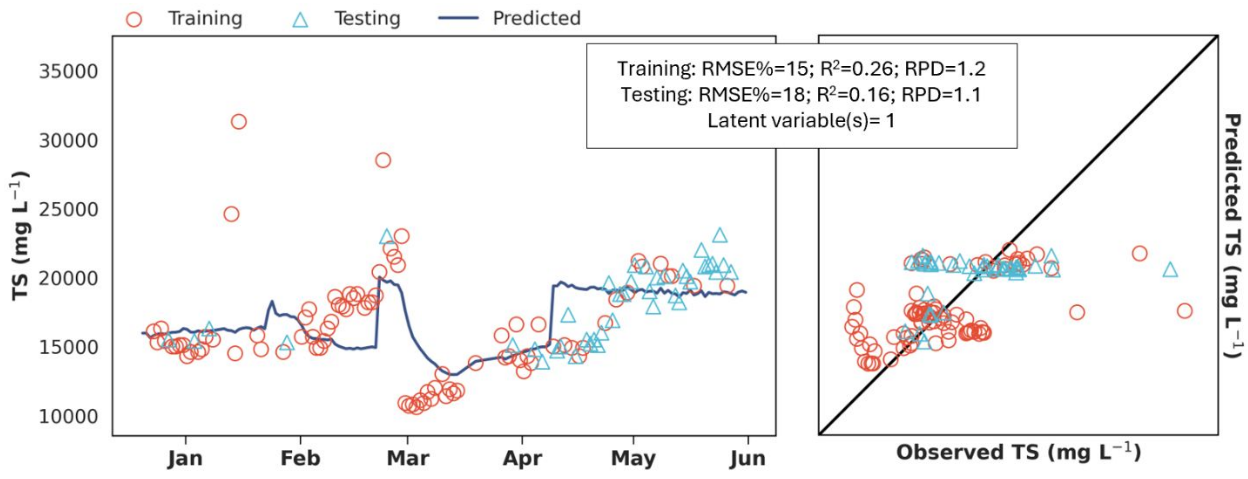

Figure S5. Predictions compared to observations of TS in digestate. Results of training and testing the model predicting TS in digestate over the sampling period (December 20, 2023, to May 31, 2024) and comparison of observations to predictions. The bisector line represents a R^2 of 1. TS, total solids; mg L^{-1}, milligrams per liter; RMSE%, percent root mean square error; R^2, correlation coefficient; RPD, residual prediction deviation.

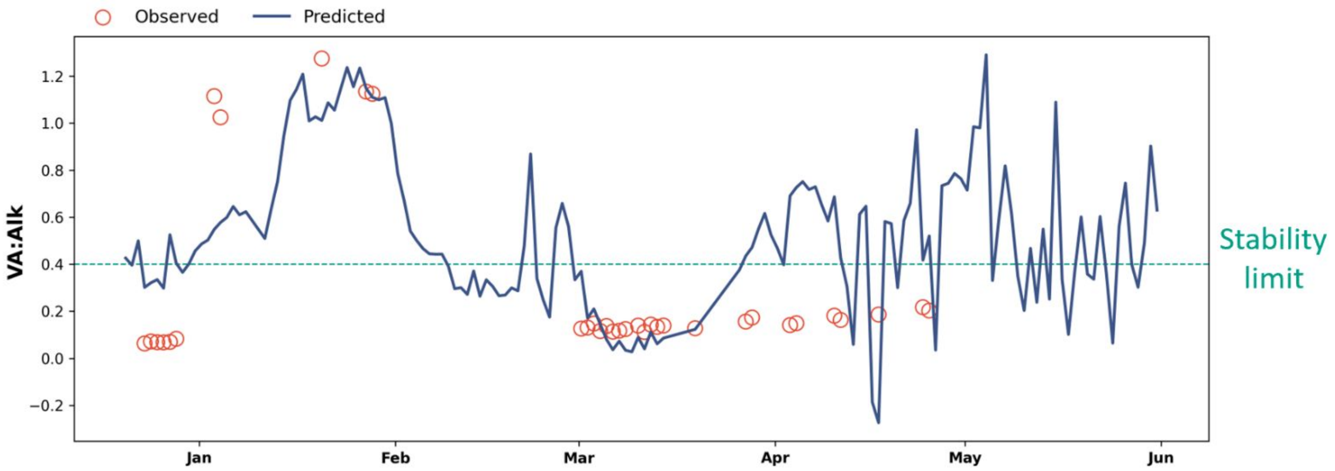

Figure S6. Predictions compared to observations of the volatile acid to alkalinity ratio in whole digestate sample (VA:Alk) over the sampling period (December 20, 2023, to May 31, 2024). Dashed horizontal line indicates an upper stability limit of 0.4.

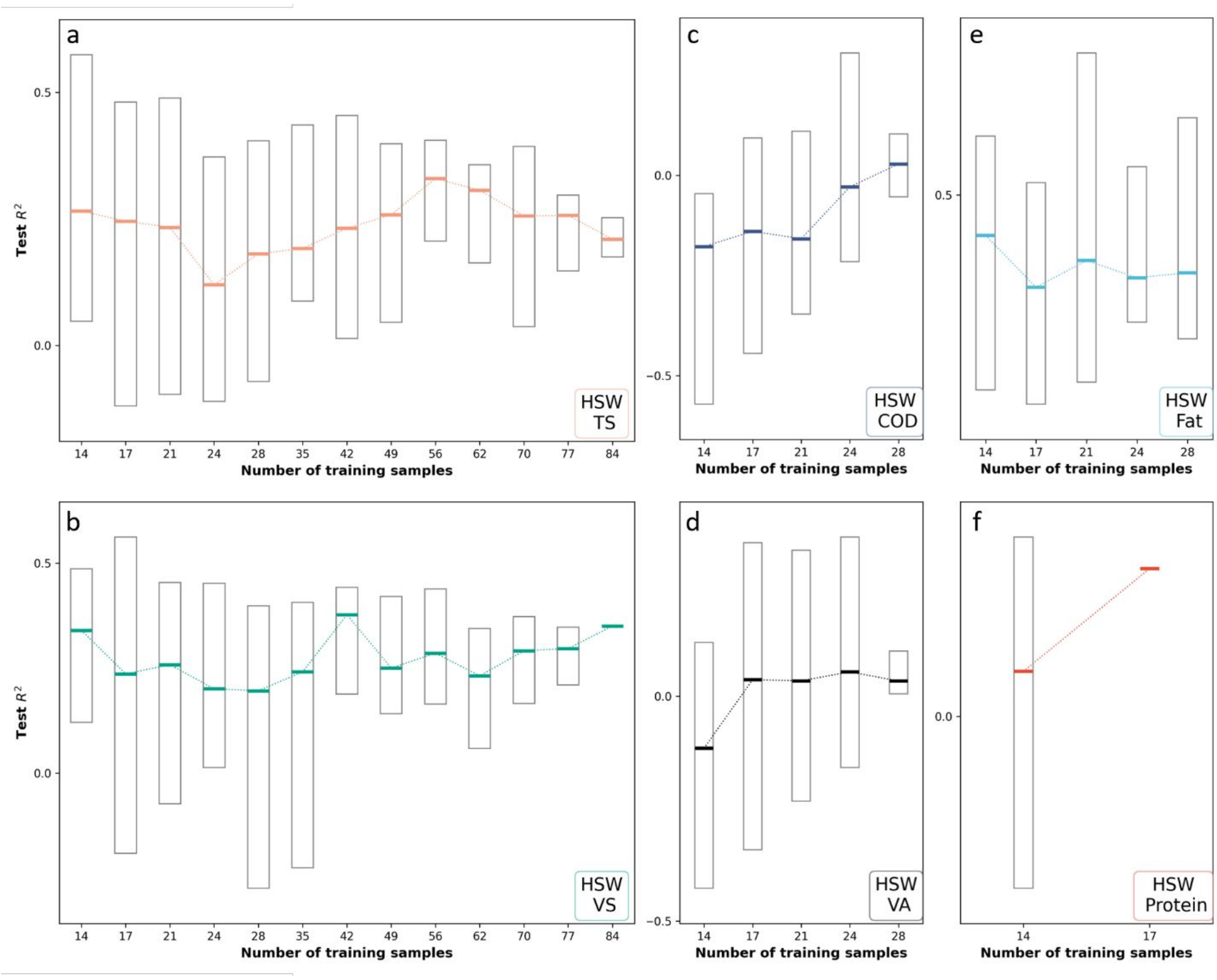

Figure S7. Summary of correlation coefficients (R^2) of HSW predicted parameters after downsampling. Each boxplot represents the distribution of R^2 after 20 repetitions using the indicated number of training samples in each model. Horizontal line indicates the median while the edges of the boxes are the first and third quartiles. R^2 summary of: (a) TS, (b) VS, (c) COD, (d) VA, (e) fat content, and (f) protein content. COD, chemical oxygen demand; HSW, high-strength waste; TS, total solids; VA, volatile acids; VS, volatile solids.

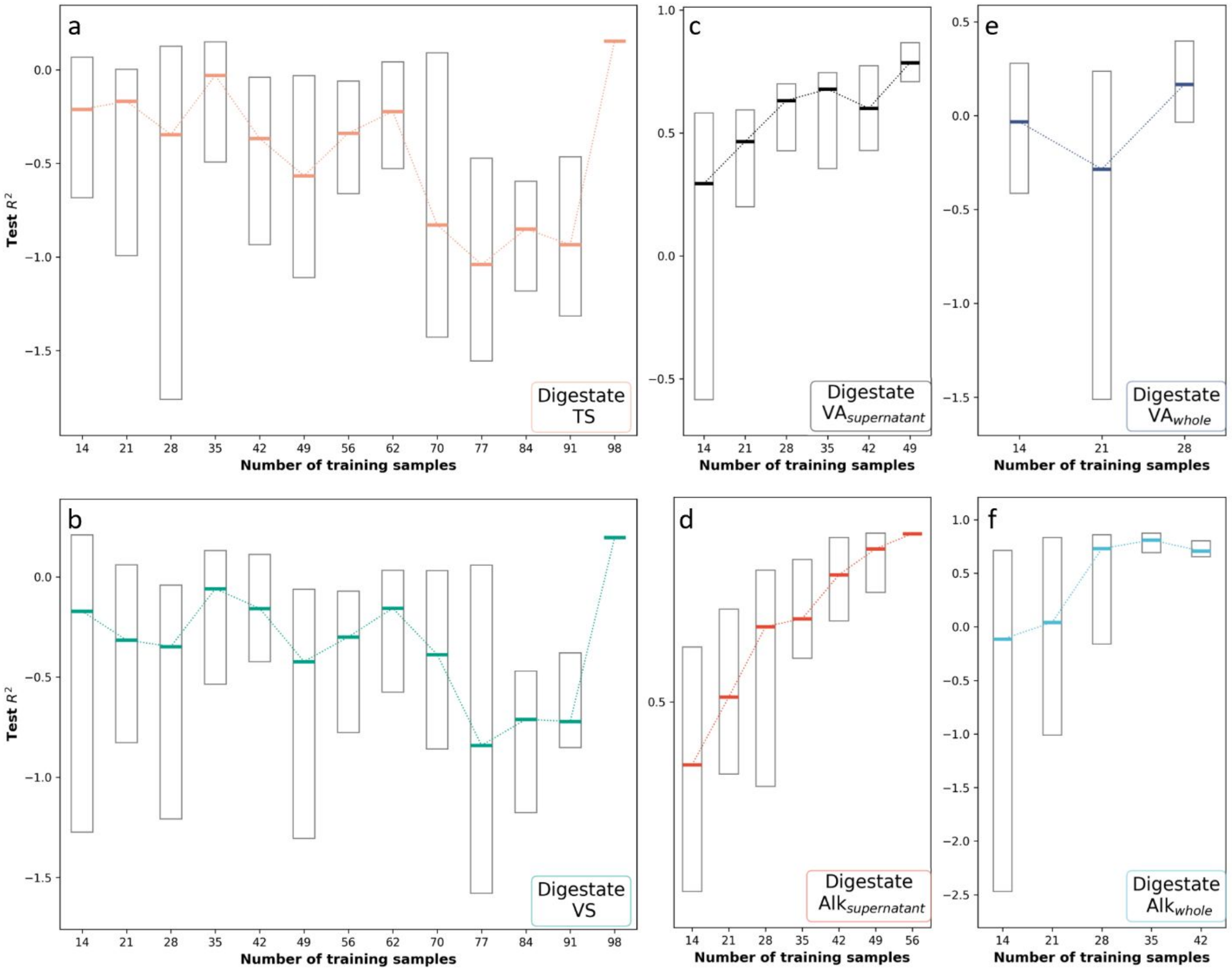

Figure S8. Summary of correlation coefficients (R^2) of digestate predicted parameters after downsampling. Each boxplot represents the distribution of R^2 after 20 repetitions using the indicated number of training samples in each model. Horizontal line indicates the median while the edges of the boxes are the first and third quartiles. R^2 summary of: (a) TS, (b) VS, (c) $VA_{supernatant}$, (d) $Alk_{supernatant}$, (e) VA_{whole}, (f) Alk_{whole}. TS, total solids; VS, volatile solids; $Alk_{supernatant}$, alkalinity from digestate supernatant; VA_{whole}, volatile acids from whole digestate sample; Alk_{whole}, alkalinity from whole digestate sample; $VA_{supernatant}$, volatile acids from digestate supernatant.

$$R^2 = 1 - \frac{\sum (Observed\ target - Predicted\ target)^2}{\sum (Observed\ target - Target\ mean)^2}$$

Equation S1. Calculation for correlation coefficient.

$$RMSE\% = \frac{\sqrt{\frac{1}{n}\sum (Observed\ target - Predicted\ target)^2}}{Target\ maximum - Target\ minimum} \times 100$$

Equation S2. Calculation for percent root mean square error.

$$RPD = \frac{Standard\ deviation\ of\ observed\ target}{\sqrt{\frac{1}{n}\sum (Observed\ target - Predicted\ target)^2}}$$

Equation S3. Calculation for residual prediction deviation.

Table S1. Literature comparison of physicochemical parameters measured using rapid DRS combined with predictive modeling in anaerobic digestion systems.

Parameters	Scale	Digestion type	Train samples	Unit	Train Min	Train Max	LV	Test R^2	Test RMSE%	Reference
TS	Full scale	Mono-digestion	35	%	27.3	55.5	2	0.94	4.3	Jacobi, et al. 2011[3]
	Lab scale	Co-digestion	42	%	4.4	6.6	1	0.97	16	Lomborg, et al. 2009[4]
VS	Lab scale	Co-digestion	65	g kg^{-1}	53.4	86.5	3	0.76	8.9	Krapf, et al. 2013[5]
	Full scale	Mono-digestion	34	%	26.2	54.4	2	0.90	5.5	Jacobi, et al. 2011[3]
	Lab scale	Co-digestion	40	%	3.2	4.5	1	0.98	6.6	Lomborg, et al. 2009[4]
	Lab scale	Co-digestion	75	g kg^{-1}	46.1	85.4	5	0.86	13	Krapf, et al. 2013[6]
VFAs	Lab scale	Co-digestion	64	g kg^{-1}	0.2	13.1	5	0.85	6.8	Krapf, et al. 2013[5]
	Lab scale	Co-digestion	40	g L^{-1}	1.3	22.3	3	0.90	7.6	Lomborg, et al. 2009[4]
	Lab scale	Co-digestion	75	g kg^{-1}	0.49	11.61	5	0.75	9.1	Krapf, et al. 2013[6]
	Full scale	Mono-digestion	131	g kg^{-1}	0.31	10.2		0.94	8.3	Jacobi, et al. 2009[7]
	Lab scale	Co-digestion	38	g L^{-1}	4	36	4	0.96	12.9	Holm-Nielsen, et al. 2008[8]
	Lab scale	Mono-digestion	53	mg L^{-1}	1084	11112	4	0.92	17.1	Holm-Nielsen, et al. 2011[9]
Alkalinity	Lab scale	Co-digestion	153	g HCO_3 L^{-1}	0	22.4	10	0.54		Ward, et al. 2011[10]
	Lab scale	Co-digestion	153	g HCO_3 L^{-1}	0	22.4	13	0.36		Ward, et al. 2011[10]
Fat	Full scale	Mono-digestion	35	%	0.55	1.87	4	0.70	6.8	Jacobi, et al. 2011[3]
Protein	Full scale	Mono-digestion	35	%	2.05	4.11	3	0.72	12	Jacobi, et al. 2011[3]

TS, total solids; VS, volatile solids; VFAs, volatile fatty acids; LV, latent variables; R^2, correlation coefficient; RMSE%, percent root mean square error.

Table S2. Features of datasets for training and testing the partial least squares regression model after Kennard-Stone partition.

Waste stream	Parameter	Unit	Train Samples	Test Samples	Train Min	Train Max	Train Mean	Train Std	Test Min	Test Max	Test Mean	Test Std
HSW	TS	mg L^{-1}	84	37	31600	224233	86739	39702	70900	184600	100245	40398
	VS	mg L^{-1}	84	36	22600	204270	78617	39020	46022	177216	93084	37822
	COD	mg L^{-1}	29	13	17600	591400	181400	109800	79800	384500	193200	76700
	VFA	mg L^{-1}	29	13	7790	38400	22150	7490	8470	41700	24120	8090
	Fat	% of TS	28	13	1.45	79.9	52.2	20.8	39.0	78.3	58.6	11.8
	Protein	% of TS	17	8	6.25	39.7	26.3	8.34	16.2	32.6	24.6	5.98
	Carbs	% of TS	17	8	2.32	68.6	24.7	17.6	3.18	35.9	13.9	10.5
Digestate	TS	mg L^{-1}	98	42	10600	31300	16240	3606	13900	30400	18512	3193
	VS	mg L^{-1}	98	42	8141	27732	12168	2512	10967	16811	13400	1524
	TAC	mg L^{-1}	47	21	2067	6376	3213	1274	2253	5752	2824	898
	FOS	mg L^{-1}	30	14	269	5514	1435	1662	308	5769	1520	1675
	Alk	mg L^{-1}	56	24	1811	6168	3596	1230	1844	4648	2936	748
	VA	mg L^{-1}	54	24	102	575	239	105	109	391	187	65

TS, total solids; VS, volatile solids; COD, chemical oxygen demand; VFA, volatile fatty acids; Carbs, carbohydrates; TAC, alkalinity from whole sample; FOS, volatile acids from whole sample; Alk, alkalinity from supernatant; VA, volatile acids; Min, minimum chemical value; Max, maximum chemical value; Mean, average chemical value; Std, standard deviation of chemical values.

REFERENCES

(1) Ahlm, P.; Bocklund, K.; Jordan, B.; McFarlane, D.; Zaghdoudi, M. Anaerobic Digestion Evaluation Study. *Great Plain Institute* **2018**. https://recyclingandenergy.org/wp-content/uploads/2021/01/2018-09-GPI-Anaerobic-Digestion-White-Paper-Final-Report-1.pdf (accessed 2024)

(2) United States Environmental Protection Agency. Volume-To-Weight Conversion Chart. In *RecycleMania* **2010**. https://archive.epa.gov/wastes/conserve/tools/rogo/web/pdf/volume-weight-conversions.pdf (accessed 2024)

(3) Jacobi, H. F.; Moschner, C. R.; Hartung, E. Use of near infrared spectroscopy in online-monitoring of feeding substrate quality in anaerobic digestion. *Bioresource Technology* **2011**, *102* (7), 4688-4696. DOI: 10.1016/j.biortech.2011.01.035

(4) Lomborg, C. J.; Holm-Nielsen, J. B.; Oleskowicz-Popiel, P.; Esbensen, K. H. Near infrared and acoustic chemometrics monitoring of volatile fatty acids and dry matter during co-digestion of manure and maize silage. *Bioresource Technology* **2009**, *100* (5), 1711-1719. DOI: 10.1016/j.biortech.2008.09.043

(5) Krapf, L. C.; Heuwinkel, H.; Schmidhalter, U.; Gronauer, A. The potential for online monitoring of short-term process dynamics in anaerobic digestion using near-infrared spectroscopy. *Biomass and Bioenergy* **2013**, *48*, 224-230. DOI: 10.1016/j.biombioe.2012.10.027

(6) Krapf, L. C.; Nast, D.; Gronauer, A.; Schmidhalter, U.; Heuwinkel, H. Transfer of a near infrared spectroscopy laboratory application to an online process analyser for in situ monitoring of anaerobic digestion. *Bioresource Technology* **2013**, *129*, 39-50. DOI: 10.1016/j.biortech.2012.11.027

(7) Jacobi, H. F.; Moschner, C. R.; Hartung, E. Use of near infrared spectroscopy in monitoring of volatile fatty acids in anaerobic digestion. *Water Science and Technology* **2009**, *60* (2), 339-346. DOI: 10.2166/wst.2009.345

(8) Holm-Nielsen, J. B.; Lomborg, C. J.; Oleskowicz-Popiel, P.; Esbensen, K. H. On-line near infrared monitoring of glycerol-boosted anaerobic digestion processes: Evaluation of process analytical technologies. *Biotechnology and Bioengineering* **2008**, *99* (2), 302-313. DOI: 10.1002/bit.21571

(9) Holm-Nielsen, J. B.; Esbensen, K. H. Monitoring of biogas test plants—a process analytical technology approach. *Journal of Chemometrics* **2011**, *25* (7), 357-365. DOI: 10.1002/cem.1344

(10) Ward, A. J.; Hobbs, P. J.; Holliman, P. J.; Jones, D. L. Evaluation of near infrared spectroscopy and software sensor methods for determination of total alkalinity in anaerobic digesters. *Bioresource Technology* **2011**, *102* (5), 4083-4090. DOI: 10.1016/j.biortech.2010.12.046
